# Healthy ageing and depletion of intracellular glutathione influences T cell membrane thioredoxin-1 levels and cytokine secretion

**DOI:** 10.1186/1752-153X-7-150

**Published:** 2013-09-05

**Authors:** Rita Barreto Duarte Carilho Torrao, Irundika HK Dias, Stuart J Bennett, Christopher R Dunston, Helen R Griffiths

**Affiliations:** 1 Life and Health Sciences, Aston University, Aston Triangle Birmingham B4 7ET, UK

**Keywords:** Reduced glutathione, Redox, Cysteine, Ageing, Membrane protein, Thioredoxin-1

## Abstract

**Background:**

During ageing an altered redox balance has been observed in both intracellular and extracellular compartments, primarily due to glutathione depletion and metabolic stress. Maintaining redox homeostasis is important for controlling proliferation and apoptosis in response to specific stimuli for a variety of cells. For T cells, the ability to generate specific response to antigen is dependent on the oxidation state of cell surface and cytoplasmic protein-thiols. Intracellular thiols are maintained in their reduced state by a network of redox regulating peptides, proteins and enzymes such as glutathione, thioredoxins and thioredoxin reductase. Here we have investigated whether any relationship exists between age and secreted or cell surface thioredoxin-1, intracellular glutathione concentration and T cell surface thioredoxin 1 (Trx-1) and how this is related to interleukin (IL)-2 production.

**Results:**

Healthy older adults have reduced lymphocyte surface expression and lower circulating plasma Trx-1 concentrations. Using buthionine sulfoximine to deplete intracellular glutathione in Jurkat T cells we show that cell surface Trx-1 is lowered, secretion of Trx-1 is decreased and the response to the lectin phytohaemagglutinin measured as IL-2 production is also affected. These effects are recapitulated by another glutathione depleting agent, diethylmaleate.

**Conclusion:**

Together these data suggest that a relationship exists between the intracellular redox compartment and Trx-1 proteins. Loss of lymphocyte surface Trx-1 may be a useful biomarker of healthy ageing.

## Background

During ageing, there is a progressive decline the ratio of cysteine to cystine and reduced to oxidised glutathione in the plasma which has been attributed to excessive oxidants within a proinflammatory environment [1]. While such changes can be accompanied by an increase in oxidatively damaged molecules, it is likely that oxidative damage accumulation during ageing may be more of a bystander effect than an ageing mechanism as recent studies have shown that manipulating the levels of many antioxidant genes and consequently the extent of molecular damage in a number of species does not inhibit the ageing process [2].

Several physiological systems including cells of the immune system also lose their homeostatic capacity with age [3,4]. Adaptation is a key process for the acquired immune system in order for novel antigens to be recognised and specific response developed. Recent studies have implicated a cooperative interaction between the intracellular T cell redox environment and exofacial membrane proteins, which ultimately influences T cell function in health and disease [5,6].

Cellular redox balance is achieved via three major redox couples; NAD-NADH; NADP-NADPH and the cysteine containing tripeptide, glutathione (GSH) - oxidised glutathione (GSSG) [7]. Cellular GSH concentration is dependent on the activity of gamma-glutamyl cysteinyl ligase (GCL) and cysteine availability [8]; expression of the rate limiting GCL enzyme is coupled to cellular redox state through the Nrf-2-KEAP1 system, providing a mechanism for cellular adaptation to oxidative stress through de novo GSH biosynthesis [8]. Therefore a decrease in protein thiols e.g. through oxidation should give rise to an increase in de novo GSH synthesis so that cellular redox state is restored.

T cell protein-thiol oxidation may arise from many processes including; 1) an increase in reactive oxygen/nitrogen species production; 2) lack of free thiols on amino acids, peptides and small proteins which serve a scavenging function e.g. cysteine, GSH and thioredoxin; and 3) inefficient enzymic reduction of oxidised thioredoxin or GSSG back to reduced thioredoxin and GSH. These latter processes are normally catalysed by thioredoxin and glutathione reductases which require NADPH as a cofactor [9].

An increase in intracellular oxidised GSSG can normally be minimised by promoting its efflux via multidrug resistance-associated proteins [10]. In addition, organelles such as the mitochondrion (which also express thioredoxin 2 uniquely) and the nucleus maintain active transport processes for GSH to preserve a local reducing environment against concentration gradients as required for cell proliferation, active gene transcription and to minimise damage from reactive oxygen species (ROS) leakage during respiration [9,10]. The efficiency of cytosolic thioredoxin 1 (Trx-1) is likely to be of particular significance during chronic inflammation when ROS/reactive nitrogen species (RNS) production by phagocytes will favour a more oxidising extracellular environment [11].

Trx-1 is a small, 12-kDa, conserved and ubiquitous multifunctional protein with several redox-active cysteine residues. It acts as an antioxidant, anti-inflammatory agent and redox-regulating enzyme (reduces disulphide bonds and sulphenic acids but also uniquely to Trx-1, has transnitrosylation activity) [12-15]. Trx-1 regulates chemokine activity, reduces inflammation, cellular infiltration, and lipopolysaccharide (LPS)-induced oxidative damage. Trx-1 has many interaction partners depending on its cellular localization. The most energetically and physiologically favourable reaction for Trx-1 is to reduce oxidised peroxiredoxins within the redox network [16]. Through its reductase activity it may regulate apoptosis, cell growth, differentiation, migration, angiogenesis, tumorigenesis, and development [17,18]. In the nucleus, Trx-1 binds directly to different transcription factors and thereby modulates their DNA-binding activity, eg, p53, nuclear factor-κB, and AP1 [19,20]. With respect to apoptosis inhibition, at least three binding partners have been identified in the cytoplasm; the apoptosis signaling kinase 1, the thioredoxin-interacting protein and actin, where actin protects Trx-1 from degradation and preserves its anti-apoptotic function [17,21]. Trx-1 also associates with the plasma membrane; it is trafficked with a limited number of cytosolic proteins via the leaderless secretory pathway, with anchorage in the membrane probably mediated by palmitoylation of cysteine [22]. Trx-1 may also be secreted, exerting a range of effects on T cells, B cells and fibroblasts from growth arrest to autocrine activation of T cells [23]; extracellular Trx-1 influences the redox state and function of ligands such as interleukin (IL)-4 [24] and maybe taken up by adjacent cells via lipid rafts when cysteine is oxidised [25]. The post-translational modifications to cysteine on Trx-1 appear critical to its localisation and function in a range of cells.

Normally, the ageing immune system is characterised by an inflammatory phenotype, increased risk for autoimmunity and reduced antigen-specific immune responses, a phenomenon referred to as ‘immunosenescence’. Typical dysfunction in immune responses related to cellular dysregulation, include impaired phagocytosis by neutrophils [26] and in T cells, reduced levels of TCR/CD28 receptor expression due to transcriptional inactivation [27] and skewing of immune effector pathways by persistent pathogens such as cytomegalovirus (CMV) that stimulate futile clonal expansion and senescence [28]. Consequently, ageing T cells are considered to be hyporesponsive to stimulus and refractory to apoptosis, a phenomenon that we have previously associated with altered redox state [11].

We have previously reported that loss in intracellular GSH during hypoxia enhanced T cell interleukin 2 receptor expression in response to phytohaemagglutinin (PHA) and that cytotoxic effects of methotrexate were decreased [29]. Using plumbagin, a thiol depleting agent that increases cytosolic ROS, mitogen-induced T-cell proliferation and cytokines (interleukin (IL)-2/IL-4/IL-6/interferon-gamma) production was suppressed and this effect was reversed by thiol antioxidants but not by non-thiol antioxidants [30]. Buthionine sulfoximine (BSO), an inhibitor of GSH synthesis, markedly reduced T cell proliferation without affecting viability and blocked production of IL-2 and IL-6 [31]. In contrast, others have shown that BSO could not inhibit IL-2 production i.e. lymphocyte activation but did inhibit cell cycle entry and proliferation [32,33]. Indeed exogenous GSH has been shown to inhibit IL-2 synthesis in mitogenically stimulated T cells although was required for DNA synthesis by Roth and Droge [34], but in contrast exogenous GSH decreases IL-4 but not IL-2 production in peripheral blood lymphocytes [35].

Here we have investigated whether ageing affects the exofacial distribution of Trx-1 on T cells, its secretion into the plasma or cell culture media and whether this change is brought about by the anticipated change in redox state observed in ageing. Improved understanding of any changes in expression or distribution of Trx-1 might improve our understanding of T cell responses during ageing and may prove to be a useful biomarker of the ageing process. We show that membrane Trx-1 and soluble plasma Trx-1 levels are decreased during healthy ageing and decreased on Jurkat T cells after inhibition of glutathione synthesis.

## Results

Trx-1 is a small protein with antioxidant and regulatory functions which is present at high concentrations intracellularly. It works in concert with other antioxidant enzymes and NADPH as a reducing agent to maintain cellular redox status, and it is involved in the regulation of redox signalling. It is considered pivotal for growth promotion, inflammatory modulation and has anti-apoptotic activity. Regulation of these physiological pathways declines with age and therefore we have explored the whether immune cell Trx-1 is modulated by ageing.

**Figure 1:**
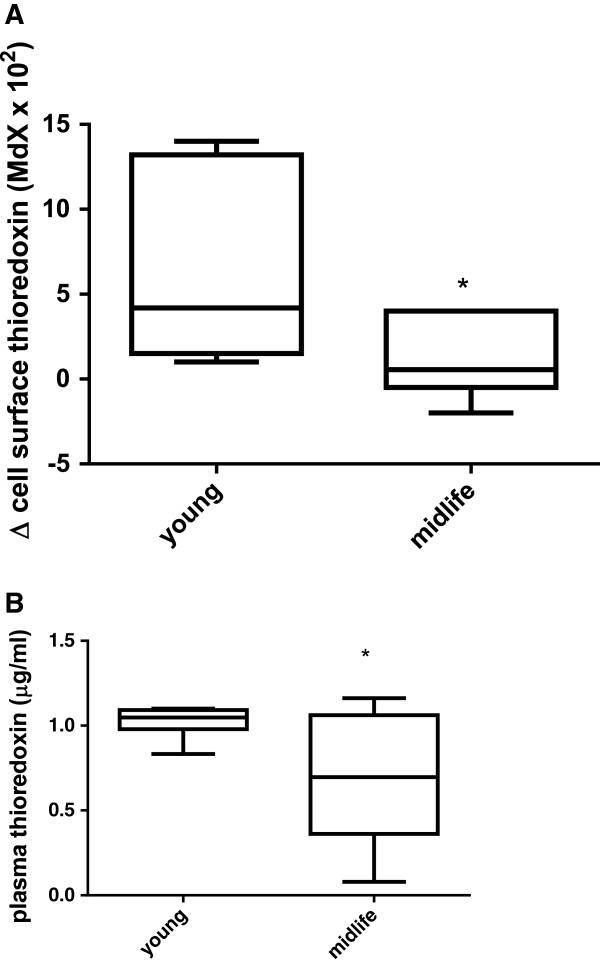
**Older adults express lower surface and secrete less Trx-1 than younger adults. (A)** Peripheral blood was collected from consenting volunteers (n = 6/group) into Optilyse prior to staining with anti-Trx-1 or isotype control antibody on ice for 30mins and detection by goat-anti-mouse APC-Cy7 conjugate. Data are expressed as the difference in MdX between antigen specific and isotype control signal over 5000 events. **(B)** Plasma Trx-1 was measured competitive inhibition ELISA. Data represents the mean +/− SEM where * represents p < 0.05 by unpaired t test with Welch’s correction for unequal variances.

**Figure 2:**
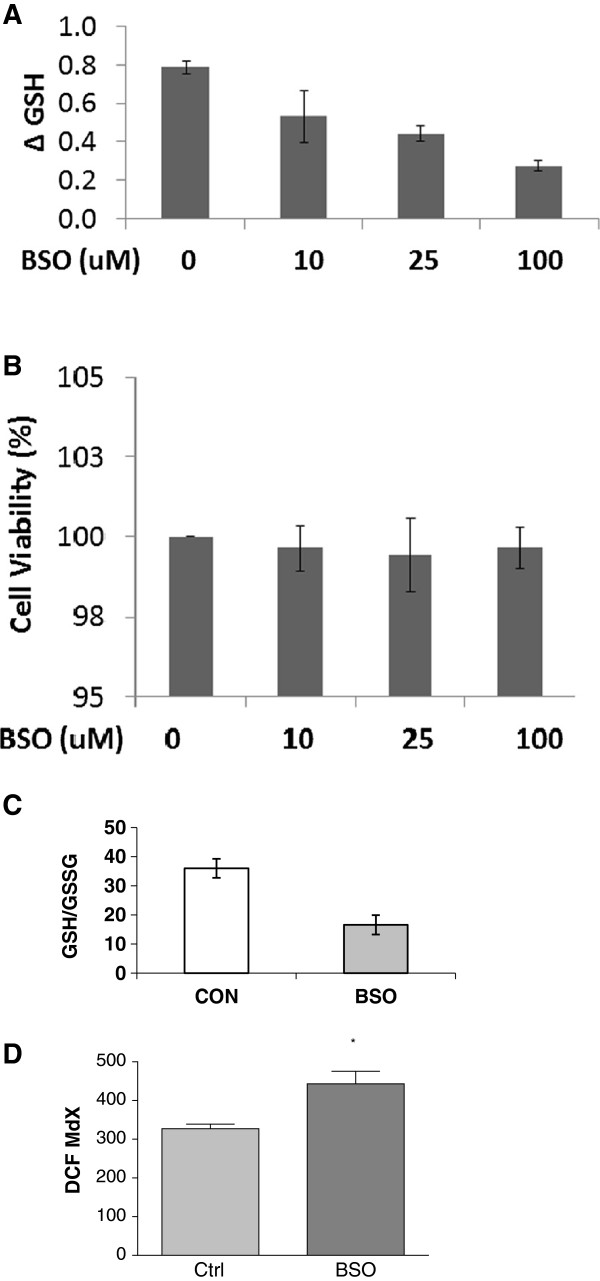
**Buthionine sulfoximine (BSO) depletes Jurkat T cell of GSH.** Jurkat T cells treated with BSO for 24 hours were depleted of intracellular glutathione as determined by the GSH recycling assay **(A)**. This was not associated with any loss in viability determined by trypan blue exclusion **(B)**. The redox ratio of reduced to oxidised GSH was decreased by 50% after incubation with 25 μM BSO for 24 hours **(C)**. The effect of BSO treatment on intracellular ROS was determined as DCF fluorescence after incubation with 25 μM BSO for 24 hours **(D)**. Data represents the mean +/− SEM of three experiments where * represents p < 0.05.

Using flow cytometry we have shown that peripheral blood lymphocytes from older adults (>50 years of age) expressed less surface Trx-1 than the lymphocytes of younger adults (mean age = 26.2 years); Figure 1A. When we permeabilised primary cells with triton x-100 after fixation, the levels of Trx-1 detected were significantly higher than in non-permeabilised cells and there were no differences between individuals according to age (data not shown). Moreover, secreted levels of Trx in the plasma were also significantly lower in older adults (Figure 1B). In order to investigate whether cellular redox stress drives a cellular adaptation in Trx-1 distribution, we used the GCL inhibitor, BSO, to deplete intracellular GSH without affecting viability in Jurkat T cells. T cell GSH was determined by the 5,5′-dithiobis-(2-nitrobenzoic acid) (DTNB) recycling assay after BSO treatment for 24 and 48 hours. Figure 2A confirms that intracellular GSH is depleted after 24 hour incubation with BSO in a dose dependent manner. Moreover, cell viability was unaffected by this treatment (Figure 2B) despite a 50% reduction in the concentration of reduced to oxidised GSH (Figure 2C). There was a slight but significant increase in intracellular peroxides associated with BSO treatment as determined by a 30% increase in dichlorofluorescein acetate fluorescence (DCF) after 24 hours (Figure 2D).

**Figure 3:**
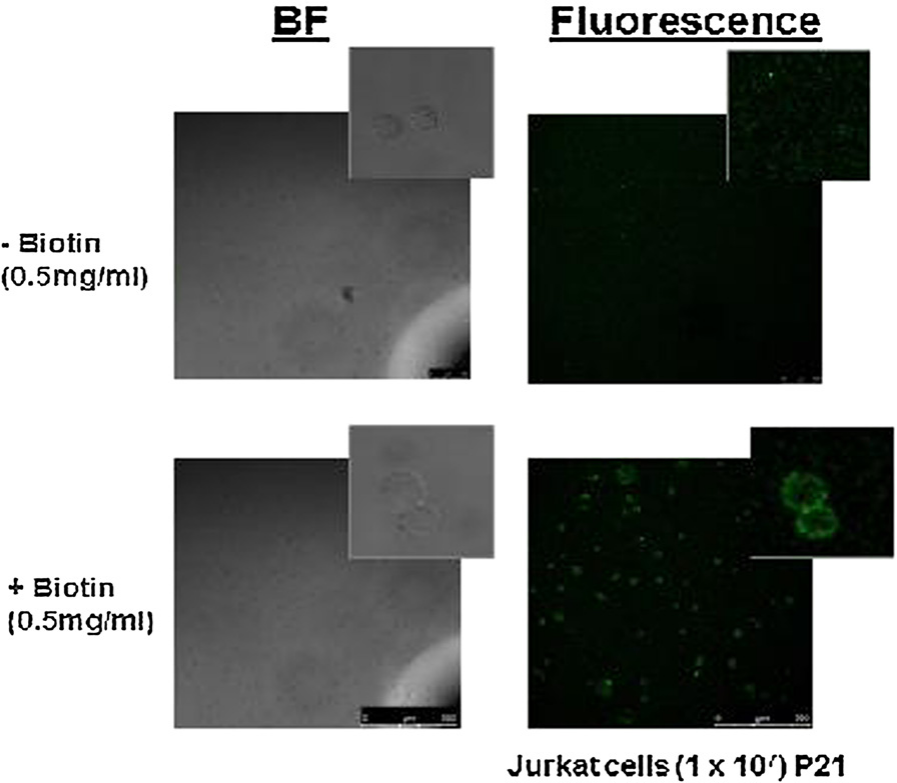
**Membrane proteins can be selectively purified from T cells by biotin capture.** Jurkat T cells were labelled NHS-S-S-biotin linker and stained with streptavidin alexafluor488. Labelling is evident on the surface of the cell only.

**Figure 4:**
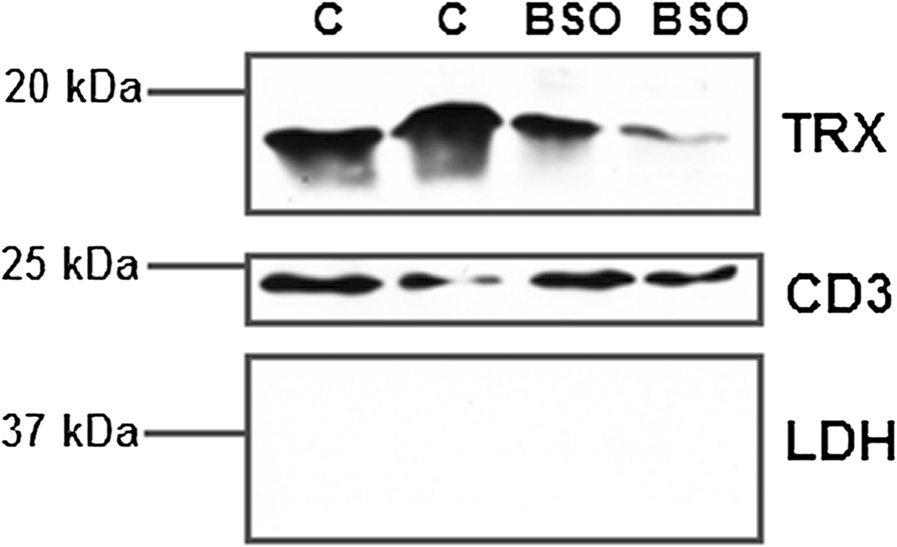
**Trx-1 distribution to the membrane is impaired by buthionine sulfoximine (BSO).** Biotinylated plasma membrane surface proteins from Jurkat T cells without (C, CON) and with (BSO) glutathione depletion, were purified by streptavidin beads and characterised using antibodies to detect plasma membrane (CD3) and cytosol (lactate dehydrogenase, LDH) proteins and thioredoxin (Trx-1).

During cell ageing and under stress, secretion of cytoplasmic proteins via the leaderless secretory pathway is altered. We used a biotin capture technique based on reaction with free amines to selectively purify membrane proteins to determine whether redox stress also exerted any effect on the translocation to the membrane and subsequent association with the extracellular face. Figure 3 illustrates that biotinylation is restricted to the surface of T cells and that streptavidin purification will capture exofacial proteins. Given that Trx-1 has been previously been described as being associated with the membrane of T cells, we investigated whether its association with the membrane is changed under oxidative stress and we studied T cell membrane Trx-1 after BSO treatment. Figure 4 illustrates that membrane-associated Trx-1 levels are lower after GSH depletion whereas CD3 expression remains unchanged.

**Figure 5:**
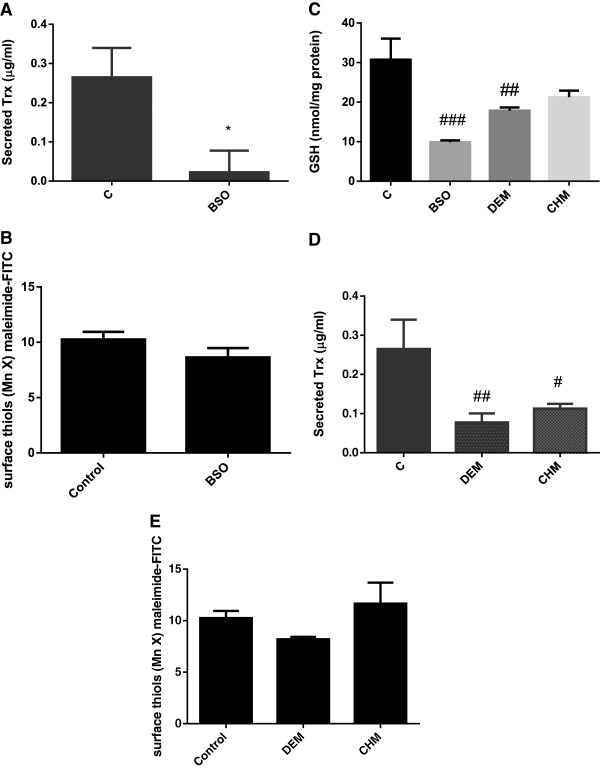
**Jurkat T cell Trx-1 secretion is reduced but surface thiol levels remain unchanged after intracellular glutathione depletion. (A)** Trx-1 secreted into the media over a 24 hour treatment with BSO (25 μM) was analysed by competition ELISA. **(B)** Surface thiols were analysed on Jurkat T cells after treatment with BSO (25 μM for 24 hours) using FITC-conjugated maleimide and analysed by flow cytometry. **(C)** Compared with buthionine sulfoximine treatment (BSO; 25 μM; 48 hours), diethylmaleate (DEM; 20 μM; 48 hours) and cycloheximide (CHM; 0.1 μg/ml; 48 hours) also depleted intracellular glutathione as determined by the DTNB recycling assay. Intracellular GSH loss was associated with a decrease in Trx-1 secretion determined by competition ELISA **(D)** but surface thiols analysed bby FITC-maleimide labelling and flow cytometry remained unaffected **(E)**. Data represents the mean +/− SEM where * represents p < 0.05 by unpaired t test with Welch’s correction for unequal variances or # represents p < 0.05, ## p < 0.01 and ### p < 0.001 by ANOVA.

To explore whether the depletion in surface Trx-1 with BSO treatment could be attributed to an increased rate of shedding or secretion of the protein, soluble Trx-1 was measured in the supernatant after 24 h incubation with BSO. Secreted Trx-1 levels were also significantly reduced by BSO treatment (Figure 5A). Despite the loss of surface and secreted Trx, there was only a small non-significant loss of cell surface thiols in the presence of BSO (Figure 5B). To investigate the robustness of the association between intracellular glutathione loss and the loss of extracellular Trx-1 we used diethyl maleate, which conjugates and depletes GSH but does not affect its synthesis, and a global protein synthesis inhibitor cycloheximide (CHM) at non-toxic concentrations. In common with BSO, these agents also depleted intracellular GSH, reduced the extent of Trx-1 secretion and had no effect on extracellular thiol content (Figures 5C-E).

**Figure 6:**
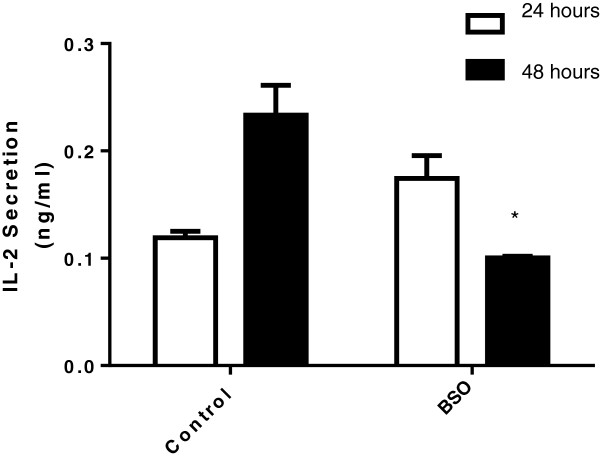
**Glutathione (GSH) depletion by buthionine sulfoximine (BSO) impairs IL-2 secretion from Jurkat T cells.** Depletion of intracellular GSH in Jurkat T cells by treated with 100 μM BSO for 24 hours reduces phytohaemaglutinin (PHA; 1 μg/ml; 24 and 48 hours) induced secretion of IL-2. Data are mean+/− SEM from three experiments, where * represents p < 0.05.

Others have shown previously that a thiol depleting agent (plumbagin) results in increased mitogen-induced T-cell proliferation but that cytokine (IL-2/IL-4/IL-6/IFN-gamma) production was suppressed. To investigate whether loss of cellular GSH specifically rather than thiols generally is an important target for altering cytokine secretion profile, we evaluated IL-2 production in Jurkat T cells in the presence of BSO. Figure 6 confirms that IL-2 secretion from T cells is reduced in response to PHA when GSH is depleted.

## Discussion

We have investigated surface Trx-1 in peripheral blood lymphocytes from healthy adults of different ages and observed that older adults express lower surface Trx-1 and secrete less Trx-1 into their plasma. However, there was no difference between young and older adult total (intra- and exofacial) expression of Trx-1.

Some cytosolic proteins are exported via a non-canonical leaderless sequence secretory pathway which can be p53 dependent [36,37]. Moreover, a major hallmark of cellular senescence is induction of the senescent phenotype which is characterized by secretion of pro-inflammatory factors; thus the condition has been called the senescence-associated secretory phenotype [38]. Given the close redox relationship between intracellular GSH and Trx-1 [9] which is transported through this non-canonical leaderless sequence secretory pathway, we have investigated whether GSH depletion influences Trx-1 localisation at the membrane.

Our present study demonstrates that a change in concentration of the major intracellular redox buffer, GSH, influences the expression of a specific redox active protein on the T cell surface, Trx-1, its secretion into the extracellular media and the response to mitogen as measured by export of IL-2; there is less Trx-1 associated with the membrane and less in the extracellular space when intracellular GSH levels are depleted. However, in considering global surface thiols on BSO-treated cells we observed that these were not affected by BSO, DEM or CHM treatment in cell culture. These findings suggest either that the stress of cell culture exceeds the redox stress applied, that all the readily oxidisable thiols exist in an oxidised form in culture or that other processes unaffected by intracellular GSH depletion maintain the surface thiol redox state.

We have shown here that Trx-1 secretion is decreased rather than increased and that total expression of Trx-1 does not appear to be decreased based on equivalent levels total Trx-1 analysed in permeabilised cells. However, it is not clear whether its rate of degradation is increased or if trafficking is affected. Others have shown that Trx-1 on the T cell surface changes during chronic disease and stress, and that the full length isoform is anti-inflammatory but the 10 kDa truncated form is pro-inflammatory [39-41]. The extracellular function of Trx-1 is suggested to be as a reducing agent. This function is also shared with protein disulphide isomerase, the activity of which is important for HIV entry, although target proteins may be discrete between the two reducing proteins [42,43]. In either case, for the enzymes to maintain their reducing activity, they require a source of reducing agents and for Trx-1, the presence of Trx-1 reductase [13]. During ageing when the extracellular environment is more oxidising the potential to regenerate the chemically reduced from of Trx-1 is likely to be lower [1]. It remains to be determined whether any proteins partner uniquely with Trx-1 and if their oxidation state is altered during ageing or following intracellular GSH depletion. Normally, human Tregs express and secrete higher levels of Trx-1 than other T cells. This may prevent uncontrolled immune reactions by favouring survival of suppressor rather than effector cells [5]. Whether the depletion of Trx-1 on T cells from older adults may predispose to less suppression of the immune response e.g. to CMV or autoantigens remains to be explored [27,28].

The IL-2 secretory response to PHA follows from lectin-mediated cross-linking of surface proteins into large rafts and requires activation of either NFAT or AP1 transcription factors in the nucleus, both of which are redox dependent [44,45]. After translation, IL-2 is processed for secretion through conventional secretory pathways involving vesicular transport via the golgi. It is unknown whether the effect of GSH loss on cytokine responses observed here is most profound on IL-2 secretion or whether other cytokines are affected. However, the findings of lower response to PHA from BSO-treated Jurkat T cells are consistent with the refractory nature of older adult T cells to mitogenic stimuli which associates with oxidative stress [11]. It is not anticipated that redistribution of Trx-1 away from the surface of cells to the cytoplasm per se will influence an extracellular response directly although, it may play a more important role in lipid raft organisation and receptor clustering when internalised [46]. Instead, in the extracellular space, its capacity to play a regulatory role through catalysing the chemical reduction of receptors or ligands may be impeded.

The importance of Trx-1 in ageing has been implicated by studies that show embryonic lethality after Trx-1 knockout but enhanced lifespan for Trx-1 transgenic animals [2,47]. These findings contrast with other studies on mitochondrial Trx-2 which after overexpression, did not affect lifespan. We are now investigating the distribution of Trx-1 on mononuclear cells during ageing as part of the MARKAGE study. If the findings in our pilot study of healthy older adults and observations with GSH-depleted Jurkat cells depleted are upheld in primary cells, we will explore the role of Trx and its oxidation state on the surface of ageing T cells. Taken together we suggest that the interaction between redox state and adaptation within and on the surface of cells of ageing T cells merits investigation in healthy ageing.

## Conclusion

We have shown for the first time that the cellular distribution of Trx-1 on lymphocytes changes during healthy ageing with lower secretory and exofacial Trx-1 expression. Moreover, depletion of intracellular glutathione can recapitulate these effects in Jurkat T cells. Any direct effects of Trx-1 redistribution for cellular function, independent of GSH depletion, remain to be determined within the context of ageing.

## Experimental

### Recruitment of participants

Young male adults (18–35 years old) and mid-life male adults (50–70 years old) who were healthy, non-smokers and were not taking any disease modifying or anti-inflammatory medication or nutritional supplements were recruited. Participants provided informed written consent and ethical approval was obtained from the Aston University Ethics Committee. After an overnight fast, 5 mL whole blood was drawn from the anticubital vein of each participant and collected into ethylenediaminetetraacetic acid (EDTA) coated tubes (Greiner Bio-One Ltd, UK) between 8:00 and 10:30 am.

### Cell culture

Human Jurkat T cells from ATCC were maintained in RPMI 1640 media containing 10% foetal bovine serum and 200 U/ml penicillin and streptomycin at 37°C in a humidified atmosphere of 5% CO_2_ and 95% air. Cells were passaged at confluence and used between passages 20 and 30.

### Intracellular glutathione determination (DTNB recycling assay)

After 24 hours incubation with buthionine sulfoximine (BSO), diethyl maleate (DEM) or cycloheximide (CHM), treated cells and non-treated control T cells (5 × 10^5^ cells) were pelleted, washed twice with PBS and the pellet was air dried for 5 min. Sulfosalicylic acid (SSA; 3.33 μl of 100% made up in distilled water) was then added to the cell pellet, vortexed and immediately centrifuged at 6600 × g for 1.5 min. Stock buffer (96.6 μl of 125 mM sodium phosphate, 6.3 mM disodium EDTA, pH 7.5) was then added to each tube, vortexed and re-centrifuged as above. Supernatants were collected into fresh tubes and GSH and GSSG levels were assessed by the GSR-DTNB recycling assay on the same day or samples were immediately stored at -80°C for analysis within one month [48]. Protein concentration was measured by bicinchoninic assay [49].

### Determination of intracellular ROS

Cells were loaded with 50 μM DCFH-DA per 2 × 10^6^ cells for the final 40 minutes of BSO treatment [50]. Immediately following agent/DCFDA incubation, cells were analysed by flow cytometry (EPICS® XL-MCL), with the first control population always adjusted to the third log decade, giving a MdX value of ~100. The viable cell population, determined by FS and SS properties, were gated to exclude debris, clumped cells or machine noise. 10,000 cells were examined from each sample on a histogram of log FL1 (DCF fluorescence) versus count.

### Membrane protein preparation

Jurkat T cells (10^7^/mL) were washed three times in ice cold PBS (1 mL; pH 8) and cell surface proteins labeled with 0.5 mg/mL biotin (EZ-Link™ Sulfo-NHS-SS-Biotin, Thermo Scientific, UK) for 20 minutes at 4°C on a rotary mixer according to Zhou et al. [51]. This reagent reacts with amines and so its efficiency is not affected by the treatments used here. It includes a disulfide bond in spacer arm allows the biotin label to be removed using reducing agents such as DTT and which prevents intracellular protein capture due to the strongly reducing environment of the cell.

Cells were lysed on ice for 30 minutes in MNE lysis buffer (150 mM NaCl, 2 mM EDTA, 25 mM MES, 1 mM Na_3_VO_4_, 1% Triton X-100 and 0.1% protease inhibitor cocktail), sheared using a 21 G needle (Terumo, UK) and centrifuged at 4,500 g for 5 minutes to obtain a post nuclear supernatant (PNS). Biotinylated membrane proteins were extracted by binding to 200 μL of pre washed Magnabind™ Streptavidin beads (Thermo Scientific, UK) and eluted into extraction buffer (8 M Urea, 2 M Thiourea, 2% w/v CHAPS and 1% destreak) for SDS-PAGE analysis and Western blotting.

### Confocal microscopy

Jurkat T cells were biotinylated as described above or left unlabeled, washed three times with PBS and allowed to adhere to poly-L-lysine coated microscope slides (VWR, UK) for 20 minutes. Cells were subsequently fixed with 1% formaldehyde, 1% BSA in PBS at room temperature, rinsed in PBS and incubated with a 1:1000 dilution of 2 mg/mL streptavidin-Alexa 488 (Life Technologies, UK) for 30 minutes at 4°C. Cells were rinsed in PBS and visualized using Leica Confocal Microscopy (Leica, UK).

### Western blot for thioredoxin-1 (Trx-1)

For immunodetection of membrane-associated proteins, 15 μg of cell lysate in modified Laemmli buffer was subjected to 10% SDS-PAGE, transferred onto PVDF membrane, and blocked overnight with 3% w/v BSA in Tris buffered saline supplemented with 0.05% Tween20 [48]. The membrane was probed with primary monoclonal anti-Trx-1 (full length, 1:1000, Abcam, UK) for 2 hours at room temperature followed by extensive washing then incubated with horse radish peroxidase-labelled anti-mouse IgG (1:20000) for 2 hours. The immunoreactive bands were detected by enhanced chemiluminescence (GE Healthcare, UK).

### IL-2 analysis

After depletion of intracellular GSH for 24 hours by treatment with BSO (100 μM) cells were washed twice with PBS, resuspended in fresh culture media and stimulated with 1 μg/ml PHA-L for 24 or 48 hours. Following the cell stimulations cell culture media was collected and cells were pelleted by centrifugation (200 g, 10mins), cell free media containing secreted cytokines was stored at −20°C until analysis for IL-2 by ELISA (Peprotech, UK).

### Cell surface thioredoxin by flow cytometry

For flow cytometric analysis of Trx-1, whole blood (50 μL) was fixed by addition of 500 μL OptiLyse C (Beckman Coulter) for 2 hours and then stored at −80°C prior to analysis. Peripheral blood leukocytes were washed four times in cold wash buffer (PBS supplemented with 1% w/v BSA) and left on ice in blocking buffer (0.3 M glycine, 1% w/v BSA and 10% w/v goat serum (PAA) in PBS) for 30 minutes. Following two washes in cold wash buffer, cells were incubated with mouse monoclonal anti-thioredoxin (ab16965; AbCam) or IgG2b isotype control antibody (ab91366; AbCam) on ice for 30 minutes. After a further two washes, cells were incubated with goat anti-mouse polyclonal conjugated APC-Cy7 (ab130791; AbCam) antibody on ice for 30 minutes. Finally, cells were washed twice in cold wash buffer and analysed on a Cytomics FC 500 flow cytometer (Beckman Coulter, Wycombe, UK).

### Flow cytometric analysis of cell surface thiols

Jurkat cells (1 × 10^5^) were treated with 10 μM maleimide and Alexa Fluor® 488 C5 maleimide dye (Life Technologies, Carlsbad, CA) (to the ratio of 9:1) for 30 min. Cells were washed with PBS three times and analysed free surface thiol groups by flow cytometry (Beckman Coulter).

### Thioredoxin competition ELISA

Trx-1 (2 μg/ml) 50 μl/well was applied to a Maxisorp microtiter 96 well plates (Nunc) in carbonate buffer pH9.6 and incubated for 1 hour at 37°C. Microtiter plate wells were washed 3 times with 250 μl of phosphate buffered saline containing 0.05% Tween-20, w/v (PBST) and were gently tapped on absorbent tissue paper. Following washing, nonspecific sites were blocked by adding Tween-20 1% v/v in PBS, 200 μl/well for overnight at +4°C. Standard curve (5 μg/ml-0 ug/ml) was prepared using human Trx-1 protein (ab51064; AbCam); 25 μl/well). Plasma samples or cell supernatants (25 μl/well) were plated in triplicates. Mouse monoclonal anti-hTRX-1 (25 μl of 0.5 μg/ml in PBS, ab16965; AbCam) was added to all standards and sample microtiter wells and plates were incubated at 37°C for 2 h. Following incubation, wells were washed with PBST (250 μl) 3 times and 50 μl of peroxidase conjugated anti-mouse antibody (0.2 μg/ml in PBS) was added to each well. The plate was incubated at 37°C for 2 h. Following washing 3 times with PBST (250 μl), and 200 μl of substrate solution containing o-phenylenediamine and hydrogen peroxide in 0.15 M citrate-phosphate buffer, was added. During incubation at room temperature, colour development was observed from 2 to 10 min. The colour reaction was stopped with the addition of 2 M sulphuric acid (50 μl/well). Absorbance was measured at 490 nm in a microplate reader (Bio Tek, UK).

## Abbreviations

BSA: Bovine serum albumin; BSO: Buthionine sulfoximine; CHM: Cycloheximide; DCF: Dichlorofluorescein; DEM: Diethyl maleate; DTNB: Dithiobis-(2-nitrobenzoic acid); GSH: Glutathione; GCL: Gamma-glutamyl cysteinyl ligase; GSSG: Oxidised glutathione; IL: Interleukin; LPS: Lipopolysaccharide; PBS: Phosphate buffered saline; RNS: Reactive nitrogen species; ROS: Reactive oxygen species; SDS-PAGE: Sodium dodecyl sulphate; Trx-1: Thioredoxin 1.

## Competing interests

The authors declare that they have no competing interests.

## Authors’ contributions

The authors have all made substantial contributions to conception and design, or acquisition of data, or analysis and interpretation of data; 2) have been involved in drafting the manuscript or revising it critically for important intellectual content; and 3) have given approval of the version to be published. RDCT and CRD undertook cell culture, viability and cytokine analyses. HKID analysed surface thiols, cellular glutathione and secreted Trx-1. SJB analysed cell surface Trx-1 by flow cytometry and western blotting. HRG analysed data and drafted the manuscript. All authors read and approved the final manuscript.
